# Chromosomal differentiation of schistosomes: what is the message?

**DOI:** 10.3389/fgene.2014.00301

**Published:** 2014-09-08

**Authors:** Hirohisa Hirai

**Affiliations:** Primate Research Institute, Kyoto UniversityInuyama, Aichi, Japan

**Keywords:** chiasma frequency, constitutive heterochromatin, telomere squence localization, geographical distribution, schistosomes

## Abstract

As the only group of flukes with dioecism, schistosomes are unique organisms; they not only have intriguing biological and evolutionary aspects but also are responsible for major public health problems in the developing world. Schistosomiasis caused by this fluke affects approximately 210 million people in 76 countries. In order to facilitate the discovery of eradication methods for this disease, fundamental biological outcomes must be made available. Whole genome sequence data represent one such resource applicable to discovering eradication methods and measures. Herein, I describe three remarkable chromosomal changes and briefly discuss the differentiation of the Asian and African groups of this parasite taxon. Chromosomal data and evolutionary aspects will enable us to exploit genomic information for advancing schistosome studies.

## CHIASMA FREQUENCY

**FIGURE 1:**
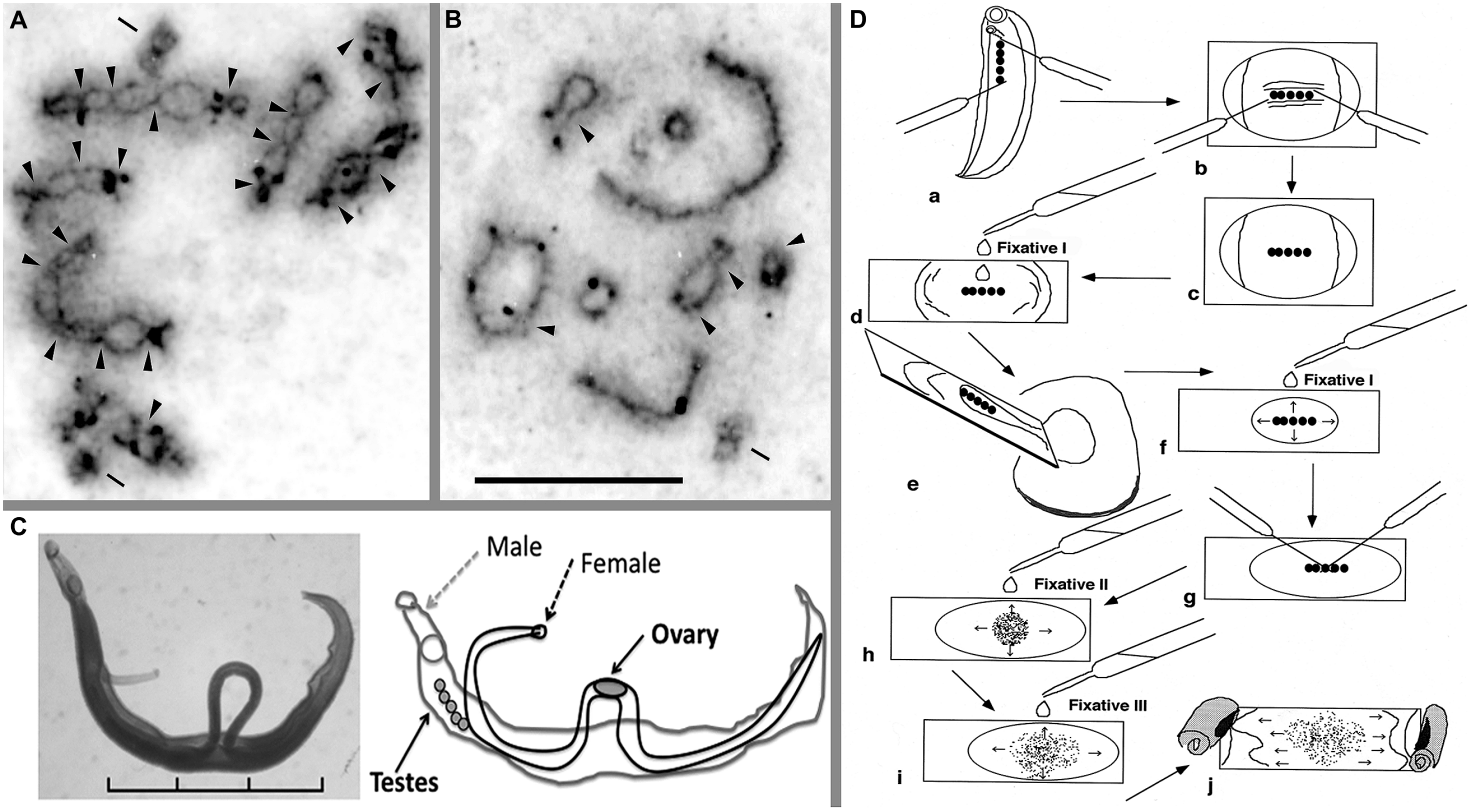
**Meiotic chromosomes, adult worms, and a technique for chromosome observation of schistosomes.** Meiotic diakinesis stained by AG for *Schistosoma mansoni* **(A)** and *S. japonicum* **(B)**. AG stain shows the synaptonemal complex (SC) protein, which detects crossing over between two homologous chromosomes. **(C)** Pairing form of the adult female and male of *S. mansoni* and the schematic illustration. **(D)** A manual preparation to obtain meiotic chromosomes from adult schistosome worms. This schema shows an example of the process with a male worm. For details, see text. Arrowhead indicates chiasma region; bar shows centriole that was stained by AG. Scale of **(A,B)** is 10 μm. Scale in **(C)** is 3 mm. See also Hirai et al. (1996, 2000) and Hirai and Hirai (2004).

When I observed the meiotic cell division of *Schistosoma japonicum* for the first time, I was amazed at the differences in the shape of the chiasmatic formation of *S. mansoni* (see **Figures 1A,B**). **Figures 1A,B** highlight the differences in the number of chiasmata between the two human schistosome species, 20 for *S. mansoni* (A) and five for *S. japonicum* (B) *S. mansoni* has several chiasmata in each chromosomal arm, but there are only a few in the chromosomal arms of *S. japonicum,* though some terminal (end-to-end) associations were observed. In our previous study, the mean frequencies of chiasmata found within arms (FXi) were 15.3 for the *S. mansoni* Puerto Rican strain and, remarkably, only 3.0 for the *S. japonicum* Japanese strain (Hirai et al., 1996). This investigation revealed a clearly different situation in chiasmatic formation between these species. In addition to this difference, Asian schistosome species showed a regional cline of FXi rate of chiasma frequency; specifically, the values for *S. japonicum* Leyte, *S. japonicum* Mindanao, *S. japonicum* Luson, *S. japonicum* Anhui (China), *S. mekongi*, and *S. malayensis* were 3.6, 7.2, 7.5, 6.3, 8.6, and 9.0, respectively (Hirai et al., 2000).

What is the biological meaning hidden in these differences in chiasmata frequency? Chiasmata are chromosomal phenotypes of crossing-over (gene shuffling) between homologous chromosomes (John, 1990). Does the difference in the rate of crossing-over inform us of any significant relationships between the gene shuffling rate and evolutionary history in schistosome species? Because these differences in the FXi were significant, there most likely are biological meanings in the difference between *S. mansoni* and *S. japonicum* and among regional races of *S. japonicum*. A method to determine what differentiates species and races would be helpful, though a viable method is not yet available.

A sophisticated technique is not necessary to observe meiotic cell division, and only two dissection needles are required. I have used the following effective procedure, which is useful for observing chiasmata of schistosomes. It is possible to observe chromosomes of small organisms like schistosomes using this technique. Only the tiny testes and ovaries (each about one millimeter in length) of the adult schistosome worms with a body length of approximately 6–26 mm (**Figure 1C**) can be used to observe meiotic chromosomes. Briefly, the order of the procedure is as follows (**Figure 1D**): (a) dissect out the testes, (b) remove other tissues, (c) treat with a hypotonic solution (0.005% colchicine in 1% sodium citrate) on a culture glass slide for 30 min at room temperature, (d) transfer the testes to and pre-fixate with Fixative I (60% acetic acid:ethanol = 1:3) on a glass slide, (e) remove surplus solution and re-fixate with Fixative I, (g) tease the testes with two needles, (h) first, spread cells with drops of Fixative II (acetic acid:ethanol = 1:1), (i) second, spread cells with Fixative III (acetic acid only) after spreading out Fixative II, and (j) finally, desiccate at room temperature. For the details of this procedure, see Hirai and Hirai (2004). This technique is useful to make dry chromosome preparations from small organs, which are then available to clearly observe several stages of meiotic cell division. We were able to utilize silver nitrate (Ag) staining to detect the synaptonemal complex (SC; **Figures 1A,B**) and fluorescence *in situ* hybridization (FISH); thus, the chromosome preparations are readily adaptable for different techniques. The comparisons of gene order and genome structure between the Z and W chromosomes using the FISH technique are very useful (Hirai et al., 1989; Spotila et al., 1989; Criscione et al., 2009). When no dried chromosome preparations were available, I could not detect differences in chiasmata among schistosome species. In particular, the chiasmata frequencies of *S. mansoni* and *S. japonicum* were quite different from each other, and Asian species showed cline gradation of change in chiasmata frequency (Hirai et al., 2000).

Genetic crossing-over and chiasma formation are chromosomal actions to recombine maternal and paternal genetic elements at the next generation. That is, the action shuﬄes gene order inherited from the ancestor of the lineage. The shuﬄe can be considered gene shuffling, which is useful to develop diversity in populations or lineages. Because crossovers and chiasmata are distributed non-randomly, it is the possibility for them to work as promotional or interference mechanisms at various levels. Differences in gene shuffling may produce the differentiation of several genetic traits.

## CONSTITUTIVE HETEROCHROMATIN (C-BAND)

**FIGURE 2:**
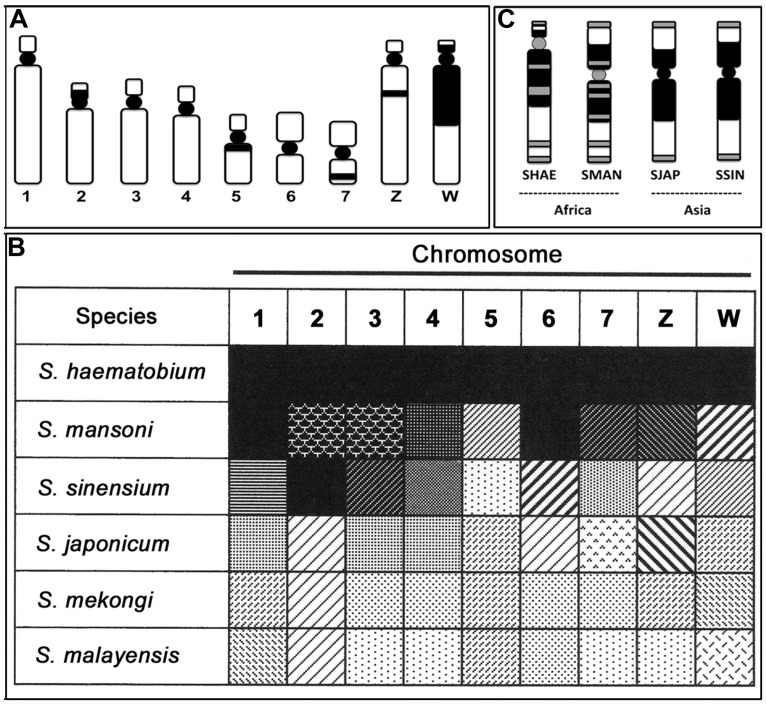
**Characteristics of constitutive heterochromatin (C-band) and telomere localizations of schistosome species. (A)** A representative C-band pattern of seven autosomes and the Z and W sex chromosomes in *S. haematobium*. **(B)** Similarity and dissimilarity of C-band patterns among six schistosome species. The black and white gradation shows the grade of resemblance, and the pattern indicates similarity of the C-band and type in each chromosome of the six schistosomes. **(C)** Localization of telomere sequence in the W chromosome of *S. haematobium* (SHAE), *S. mansoni* (SMAN), *S. japonicum* (SJAP), and *S. sinensium* (SSIN). Black represents the C-band, and gray represents the positive site to the telomere sequence in **(A,C)**. See also Hirai et al., 2000.

Mitotic chromosomes of schistosomes, which can be prepared using sporocyst stages infected in a snail host by another technique (see Hirai and Hirai, 2004), can be used with the staining method for constitutive heterochromatin (C-banding) to detect karyotypes, but not for G-banding that is available in higher order organisms like mammals. That is, G-bands are not consistently obtained as markers and are very unstable in schistosomes. Nonetheless, size, shape, and C-banding stains have therefore been used to identify chromosomes of schistosomes. I have used the TAM system (based on the non-random localization of the centromere; Imai, 1991) to classify C-banded karyotypes (see Hirai et al., 2000), which is a method to describe chromosome morphology using the existence mode of C-bands. As described in a previous paper, for example, the C-banded chromosome morphology (**Figure 2A**) of *S. haematobium* is designated as 1A^e^, 2A^ec/^, 3A^e^, 4A^e^, 5M^/c^, 6M, 7M^/i^, ZA^e/it^, and WA^ec/ci^ (Hirai et al., 2000).

Based on the representative C-band designation of *S. haematobium* chromosomes, five other species of schistosomes were also detected via karyotypes using the same nomenclature as in previous descriptions (see Figure 2 of Hirai et al., 2000). Comparing the changes in C-band patterns among six species revealed several meaningful patterns. **Figure 2B** shows this manifestation as a black-white gradation (analogy) and pattern (type of C-band). The similarity and dissimilarity of each chromosome allowed us to detect the gap and continuity of gradation patterns. Consequently, *S. haematobium* and *S. mansoni* showed higher similarity, and *S. japonicum*, *S. mekongi*, and *S. malayensis* showed similar patterns. The former and latter groups can thus be identified as African and Asian groups, respectively. In addition, *S. sinensium* appears as a mixed pattern and, therefore, is postulated to be a complex between Asian and African schistosomes.

Although chromosome paint analysis (Hirai et al., 2012) became a useful technique for identifying chromosomes of schistosomes (*S. mansoni*) in place of C-band analysis, C-banding remains valuable for investigation of chromosome evolution because the amount of heterochromatin and altered pathway of karyotypes (shape) can be ascertained only from C-band patterns. Because paint analysis is available for detecting translocation among non-homologous chromosomes, however, the combinational use of chromosome paint and C-band analyses is effective for chromosome analyses of schistosomes.

## LOCALIZATION OF TELOMERE SEQUENCE

The telomere sequence motif of schistosomes is the same as that of humans (TTAGGG; Hirai and LoVerde, 1996); the location is thus detected with a probe (CCCTAA)_7_ (Hirai et al., 2000). The localization pattern of the telomere sequence indicated specificity in the W chromosomes of four species (*S. haematobium*, *S. mansoni*, *S. japonicum*, and *S. sinensium*). Generally, tandem repeats of the telomere sequence are located at the very end of chromosomes. However, telomere sequence integration into some other regions has been observed in several species groups (e.g., Meyne et al., 1990; Go et al., 2000). Schistosome species display differences in the W chromosome between African and Asian groups. The African species showed telomere signals not only in the end regions but also in the large heterochromatin block and centromere of the W chromosome (multiple type), whereas the Asian species showed signals only in the end regions (simple type; **Figure 2C**). Accordingly, telomere sequence localization in the W chromosome also can distinguish the locality of schistosome species for African and Asian species based on the multiple and simple types of telomere localization. That is, the localization features of telomere sequences are also useful markers to determine the geographical distribution of schistosomes.

## VIEW

The study of schistosome chromosomes was pioneered by Short (1983); their work formed the basis for developing new chromosomal techniques and studies for the species, where technical challenges previously existed in the preparation of chromosome smears. We have modified the techniques for use with molecular chromosome analyses (Hirai and Hirai, 2004). Eventually, such chromosomal techniques were able to support genome sequence analyses (Berriman et al., 2009). At that point, new findings were made in the following areas: localization of repeat and female-specific DNA, telomere sequences, localization of YAC and BAC clones, gene localization, chromosome paint analysis, and evolution of the W chromosome, among others (e.g., Hirai et al., 1989, 1993, 1996, 2012; Hirai and LoVerde, 1995, 1996). Additionally, localization of molecular markers has supported genetic analyses, for example, linkage mapping (Criscione et al., 2009), which will be informative in the next generation of research on functional genomics in schistosomes. During these investigations, a surprising finding was the dramatic difference in chiasma formation among schistosome species (Hirai et al., 1996, 2000). Although the influence of this differentiation on biological manifestation remains a mystery, recently established genetic analyses (Criscione et al., 2009; Tait, 2009) have the potential to decipher the meaning and role of differences in chiasmata. Investigations related to differentiation of chiasmata will rapidly progress with application of the new genetic techniques as well as the new generation of genome sequencing. The progress made with these techniques will help advance several areas of study, including linkage disequilibrium studies, sperm typing, genome-wide patterns of recombination, detection of hotspots of recombination, and the HapMap project.

As an example of such developments, using the assumption that deletion of DNA segments is more difficult than their addition, chromosomal differentiations can indicate the direction of the change from Asian to African species groups of schistosomes. That is, African schistosomes have an insertion of a telomere sequence in some parts of the heterochromatin block and in the centromere of the W chromosome, a trait that is not observed in Asian species (**Figure 2C**). Hypothetically, deletion of all such insertions is almost impossible, even if additional insertions are possible. A hypothetical pathway of change on chromosome 2 also showed the same direction as the telomere condition (Hirai et al., 2000). If we follow this pathway, comprehensive C-band patterns also show a direction from lighter to darker gradation (**Figure 2B**). The direction of these differentiations is consistent with molecular data as well. That is, comparisons of DNA sequences strongly support that the genus *Schistosoma* originated in Asia and then distributed to Africa, indicating that there is an Asian origin for *Schistosoma* (Le et al., 2000; Attwood et al., 2002; Agatsuma, 2003). If these inferences are correct, chiasma frequency also differs from a smaller to a larger number of chiasmata from Asia to Africa, respectively (**Figures 1A,B**). Chiasma is a biologically influential mechanism of gene shuffling between homologous chromosomes in the meiotic phase and is important to ensure the maintenance of diversity in a population. A lower chiasma frequency (Asian types) results in less gene shuffling than with a larger frequency (African type). This may work to increase the breadth of biological differentiation between Asian and African schistosomes.

*S. sinensium*, described as a new schistosome species in China (Pao, 1959), is an interesting Asian species. The species seems to have derived from a common ancestor of both groups of *S. japonicum* and *S. mansoni* (Greer et al., 1989), a concept also supported by molecular analyses (Agatsuma et al., 2001). Chromosomes of *S. sinensium* also showed a mixed suite of C-band patterns (**Figure 2B**). Specifically, chromosomes 2, 3, and 4 are similar to those of *S. haematobium* and *S. mansoni* (African type), whereas chromosomes 1, 5, 7, Z, and W are similar to those of *S. japonicum*, *S. mekongi*, and *S. malayaensis* (Asian type). In addition, telomere localization in the C-band block of the W chromosome is the same as that of *S. japonicum* (**Figure 2C**). That is, this species shows a complex manifestation between Asian and African species groups not only at the morphological and molecular levels but also at the chromosomal level.

Genome sequencing in the representative three species of schistosome flukes (*S. mansoni*, *S. japonicum*, and *S. haematobium*) has opened routes to new insights and developments in biology and control of human schistosomiasis (Berriman et al., 2009; The *Schistosoma japonicum* Genome Sequencing and Functional Analysis Consortium, 2009; Young et al., 2012). These projects clarified many genes that can be used in the eradication of disease and development of drugs to control the disease. In addition, differences in the frequency of repetitive sequence were detected between the three species. The role of chiasma as an important factor in shuffling the gene structure in a genome is diminished by the existence of constitutive heterochromatin (C-band). Heterochromatin often shows species specificity because it is prone to change rapidly via the repetitive sequence, and it influences chiasma formation in many organisms (Verma, 1988). In the genome projects, the amount of repetitive elements was estimated as follows: 40% for *S. mansoni*, 40.1% for *S. japonicum*, and 43% for *S. haematobium*. We are not yet able to find direct relationships among gene characteristics, chiasma formation, amount of repetitive sequences, C-band variation, telomere sequence localization, geographical distribution, speciation, disease states, and so forth. If we apply novel generation analyses as mentioned above, however, such comprehensive biological information would undoubtedly aid in the control and even eradication of these neglected parasite diseases. For instance, determining meiotic recombination hotspots may also provide critical information for genetic differentiation, evolution, and disease control. Such sophisticated analyses are required for further studies with schistosome chromosomes and should be possible given that these approaches have already been expanded with human chromosome analyses, which are more difficult than in model organisms like mice or yeast (e.g., Egel and Lankenau, 2008).

## CONCLUSION

Until now, chiasma formation and crossing-over – genetic recombination – have not been investigated in schistosomes. They could not be previously analyzed in taxa because of the lack of methodology and technology. However, as mentioned above, such required techniques and methods are ready to be used presently. Comprehensive studies related to genetic and genomic analyses should be developed to precisely assess the features of biological interest and medically important organisms to eradicate menaces of schistosomes.

## Conflict of Interest Statement

The author declares that the research was conducted in the absence of any commercial or financial relationships that could be construed as a potential conflict of interest.
